# Clinical Profiles, Genetic Variants, and Neurodevelopmental Outcomes Following Liver Transplantation in Maple Syrup Urine Disease: A Study From Palestine

**DOI:** 10.1002/jmd2.70077

**Published:** 2026-02-22

**Authors:** Reham Khalaf‐Nazzal, Huthaifa Haj‐Ahmad, Jana Zaid, Mohammed AbuShamleh, Imad Dweikat

**Affiliations:** ^1^ Biomedical Sciences Department, Faculty of Medicine Arab American University of Palestine Jenin Palestine; ^2^ Clinical Sciences Department, Faculty of Medicine Arab American University of Palestine Jenin Palestine; ^3^ Metabolic Department Arab American University of Palestine Jenin Palestine; ^4^ Pediatric Department, Istishari Arab Hospital Ramallah Palestine

**Keywords:** living related donor liver transplantation, maple syrup urine disease, neurocognitive development and outcome, region‐specific founder variant

## Abstract

Maple syrup urine disease (MSUD) is a rare, autosomal recessive metabolic disorder resulting from a deficiency of the branched‐chain α‐ketoacid dehydrogenase complex. This leads to the accumulation of branched‐chain amino acids and their corresponding ketoacids, causing acute metabolic crises and progressive neurological damage if untreated. The impact of founder variants, high consanguinity, and limited access to metabolic care pose challenges in medically underserved populations, such as in Palestine. We conducted a retrospective analysis of 11 patients from eight Palestinian families referred to the main Metabolic Unit in the West Bank. Clinical data, biochemical profiles, and molecular findings were reviewed to characterize the presentation and outcomes of MSUD. Management strategies, including dietary intervention and liver transplantation, were also evaluated. Acute metabolic crises were the initial presentation in 91% of cases, typically within the first days of life. Diagnostic delay averaged 47 days in families without prior MSUD history, compared to 2.3 days in those with affected siblings. Founder pathogenic variants were identified in multiple unrelated families, reflecting genetic homogeneity due to community structure; novel variants were also detected. Timely diagnosis facilitated early referral and improved outcomes. Patients who underwent liver transplantation, especially when performed early, exhibited favorable developmental trajectories, increased leucine tolerance, and fewer hospitalizations. One participant diagnosed prenatally remained free of metabolic crises until transplantation at age six, with excellent neurocognitive outcomes. This study highlights the importance of integrating prenatal screening and early diagnosis, timely dietary intervention, and liver transplantation to improve MSUD outcomes in resource‐limited settings.

## Summary

- This study provides the first comprehensive clinical, biochemical, and genetic characterization of MSUD in the Palestinian population, revealing a predominance of the classical early‐onset form.
- Recurrent founder pathogenic variants in *DBT*, together with novel variants in *BCKDHA/BCKDHB*, underscore the need for population‐tailored genetic screening in high‐consanguinity settings.
- Early identification through metabolic screening, founder‐variant mapping, integrated clinical management, and timely liver transplantation was associated with markedly improved outcomes, whereas the absence of newborn screening led to delayed diagnosis, severe metabolic crises, neurological injury, and increased infant mortality.
- These findings strengthen the evidence that early liver transplantation confers substantial clinical and neurodevelopmental benefits in MSUD, particularly in resource‐limited settings with restricted access to lifelong metabolic care.

## 1 Introduction

Maple Syrup Urine Disease (MSUD) is a rare inborn error of metabolism caused by dysfunction of the branched‐chain α‐ketoacid dehydrogenase (BCKD) complex [1, 2], leading to toxic accumulation of branched‐chain amino acids (BCAA), specifically leucine, isoleucine, and valine, and their corresponding branched‐chain α‐keto acids, particularly affecting the brain [3, 4, 5, 6]. The human BCKD complex consists of four critical catalytic subunits anchored to the inner mitochondrial membrane [7, 8]. Pathogenic variants in the genes encoding these subunits disrupt BCKD function [9, 10, 11, 12, 13]. Disease‐causing variants can arise within the *BCKDHA* gene (MIM 248600) that codes the E1‐alpha subunit of the BCKD complex [14], the *BCKDHB* gene (MIM 620698) that codes the E1‐beta subunit [13], and the *DBT* gene, encoding dihydrolipoamide branched‐chain transacylase E2 (MIM 620699) [11].

Clinically, MSUD presents across a spectrum of phenotypes, ranging from the classical neonatal form characterized by early metabolic decompensation and encephalopathy to intermediate and intermittent forms with later onset [14]. Disease severity correlates with the residual activity of the BCKD enzyme complex. The classical form of MSUD, the most severe form, typically presents within the first days of life with non‐specific symptoms including poor feeding, irritability, and progressive neurological deterioration. Toxic accumulation of leucine and its metabolites cause cerebral edema and irreversible neurological damage if not promptly controlled [15]. Laboratory findings show markedly elevated plasma BCAA, often in association with ketoacidosis and hypoglycemia, reflecting profound disruption of BCAA catabolism. MSUD management primarily involves dietary restriction of BCAAs and careful monitoring of their plasma levels. Medical nutrition therapy aims to rapidly reduce the accumulation of toxic leucine metabolites while promoting anabolism and normal growth and development [16]. While medical nutrition therapy is central to treatment, management of each metabolic decompensation is complex [17]. Long‐term complications such as cognitive dysfunction and psychiatric illness often persist despite optimal therapy [17, 18]. In the classic form of MSUD, liver transplantation has shown promise in reducing metabolic crisis and facilitating long‐term metabolic control [19].

Early identification of MSUD through expanded newborn screening (ENBS) enables prompt dietary intervention and rapid reduction of plasma leucine levels at diagnosis and during metabolic crises, thereby reducing neurological risk and improving long‐term outcomes [20, 21]. The impact of ENBS was particularly pronounced in countries with high consanguinity rates, where a higher incidence of MSUD was observed and a greater number of affected infants were identified through screening [22, 23]. Currently, Palestine lacks an ENBS program. National newborn screening is limited to phenylketonuria (PKU) and congenital hypothyroidism, excluding MSUD and the majority of inborn errors of metabolism. In many affected rural communities, where consanguinity and founder variants contribute to disease prevalence, access to metabolic testing is limited, often based solely on clinical suspicion, and not provided as state‐of‐the‐art care for acutely ill children. Consequently, delays in referrals and diagnosis are common, and mortality is high when the first case presents within a family and to the healthcare system. In addition, liver transplantation is not available locally and must be accessed through specialized centers abroad, resulting in delays in definitive management and substantial logistical, financial, and emotional burdens for affected families and the healthcare system. This study provides evidence of these systemic constraints and characterizes the clinical and molecular presentation of MSUD in a cohort of 11 individuals from Palestine. Additionally, it examines the impact of timely diagnosis, dietary intervention, and liver transplantation on participants' overall health and neurodevelopmental outcomes. The study addresses gaps in the screening programs in the early recognition and management of the condition, as well as delays in treatment and care, which are made more complex by the widespread nature of the condition due to founder gene variants.

## 2 Methods

This retrospective study analyzed 11 Palestinian patients with MSUD followed over a 20‐year period at the main metabolic unit that serves the Palestinian population. This unit is staffed by a consultant metabolic specialist and supported by a pediatric specialty ward. All patients were referred from community hospitals without specialized metabolic services to this tertiary center, where care is delivered by multidisciplinary pediatric teams. Diagnostic evaluation included plasma amino acid analysis by chromatography, urine organic acid analysis, and routine laboratory investigations such as serum electrolytes, blood glucose, liver function tests, blood gas analysis, and plasma ammonia. Patient care was supported by trained pediatric nurses and specialist dietitians, with serum BCAA levels monitored every 4–6 months following stabilization during follow‐up. MSUD was diagnosed based on clinical assessment, biochemical testing, and molecular genetic analysis, with metabolic and molecular investigations performed as part of the diagnostic workup. Liver transplantation was performed at medical centers outside the country, requiring patients to travel abroad for surgical care. Immediate post‐transplantation data were obtained either through patient or family self‐report or from documented clinical assessments provided by the treating transplant centers. Sequencing of the coding and flanking intronic regions of the *BCKDHA*, *BCKDHB*, and *DBT* genes was performed in Families 1–6, while whole‐exome sequencing was used in Families 7 and 8 due to the absence of a known *DBT* founder variant in Family 7 and a first presentation with no prior family history in Family 8. Allele frequencies of identified variants were assessed using the Genome Aggregation Database (v4.1.0) [24], and variant pathogenicity was classified according to ACMG guidelines [25]. Informed consent was obtained from participants or their guardians, and the study was approved by the Arab American University Institutional Review Board (2022/C/10/N and 2024/A/3/C), in accordance with the Declaration of Helsinki and local ethical guidelines.

## 3 Results

### 3.1 Clinical Presentation

**FIGURE 1:**
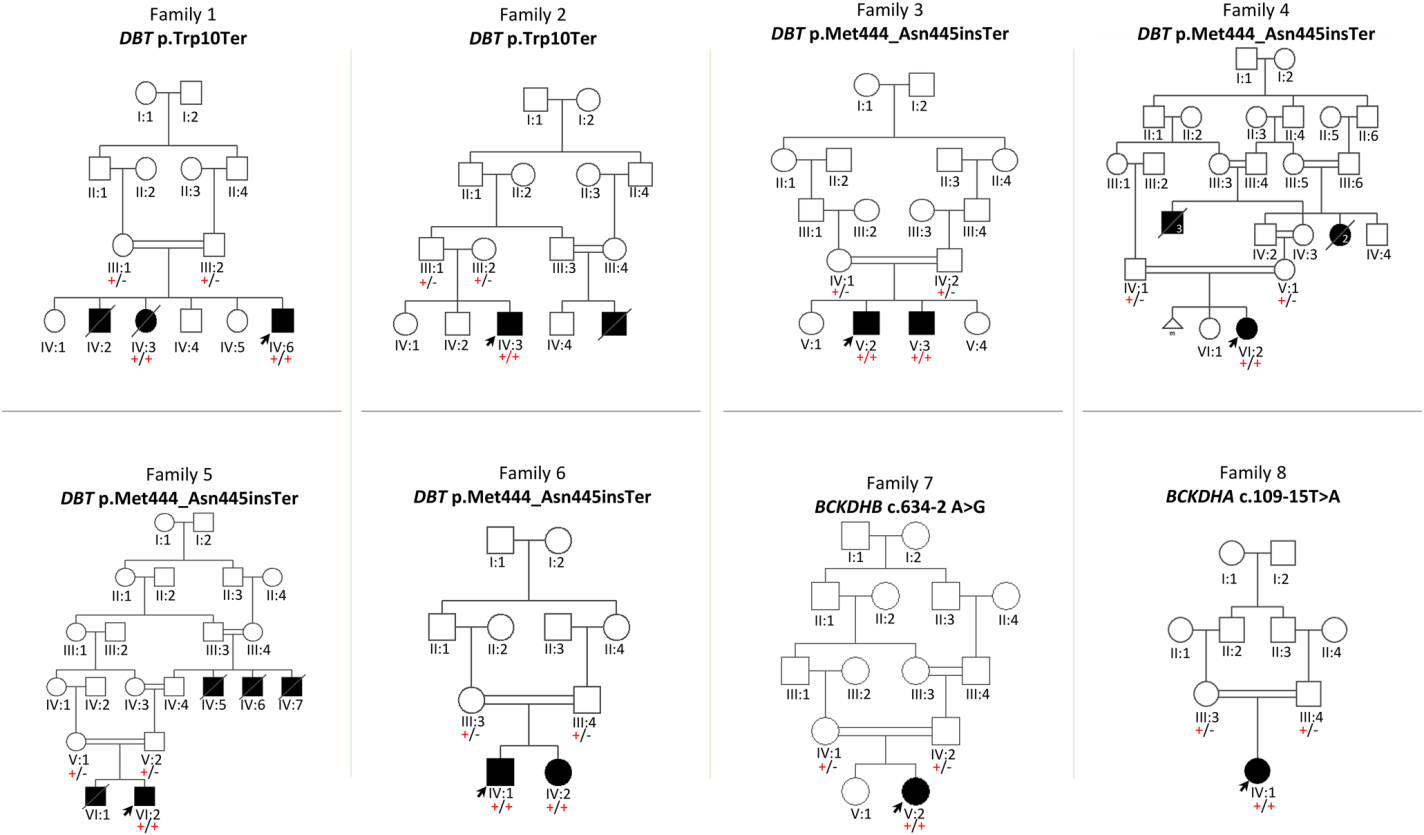
Family pedigrees and identified variants in *MSUD*‐affected families. The arrow indicates the proband in each family. Affected individuals are represented by filled squares (male) or circles (female). Filled symbols with a diagonal line denote individuals who exhibited symptoms of classical MSUD and are deceased without a definitive diagnosis. The identified gene variants are listed above each pedigree, with their segregation patterns illustrated within the families. For each individual, segregation analysis is indicated by “+” for the variant allele and “–” for the wild‐type allele.

This study involved 11 participants (six males, five females) from nine unrelated families, all diagnosed with a classical form of MSUD based on clinical presentation, metabolic testing, and molecular genetic analysis. Consanguinity was present in all families except for Family 2 (Figure 1). However, both parents in Families 1 and 2 originated from the same village, suggesting a possible distant common ancestor.

Detailed clinical histories and pedigree analyses showed that five nuclear families had multiple infants who developed early postnatal lethargy and encephalopathy, leading to the death of 12 infants within the first month of life (Figure 1, Families 1, 2, 4, and 5). Metabolic screening and genetic testing were not performed for these infants due to limited access to diagnostic services, except in Case IV:3 from Family 1 and VI:1 from Family 5.

Most participants (81%) exhibited the classical MSUD phenotype, with poor feeding and lethargy in the first few days of life progressing to encephalopathy within the first month (Table 1). Two participants (IV:6 from Family 1 and V:3 from Family 3) were diagnosed prenatally through targeted molecular testing after confirmation of the familial pathogenic variant in older siblings. This prenatal diagnosis enabled planned delivery at a tertiary care center with a multidisciplinary metabolic team and immediate initiation of dietary management after birth, resulting in sustained metabolic stability and either delayed onset of acute metabolic decompensation or, in the latter case, no decompensation at all. The most common symptoms at presentation were irritability, poor feeding, recurrent vomiting, hypotonia, and lethargy, the early indicators of classical MSUD. At diagnosis, leucine levels ranged from 552 to 4000 μmol/L (normal range 35–270 μmol/L). Seizures accompanied acute metabolic crisis in three participants (25%), individual IV:3 from Family 2, V:2 from Family 3, and the participant from Family 9, but none required long‐term medication. In the longer term, all participants experienced recurrent episodes of metabolic decompensation except for V:3 from Family 3, who was diagnosed prenatally, and was tightly compliant on the diet therapy protocol. Overall, 54.5% of participants experienced significant developmental delays and cognitive impairments, ranging from learning difficulties to moderate intellectual disability, as well as motor and speech impairments.

**TABLE 1:** Clinical and laboratory characteristics of individuals with MSUD.

Patient	Family 1, IV:6	Family 2, IV:3	Family 3, V:2	Family 3, V:3	Family 4, VI:2	Family 5, VI:2	Family 6, IV:1	Family 6, IV:2	Family 7, V:2	Family 8, IV:1	Family 9
Age at presentation (days)	120	4	3	Never	7	4	10	7	5	4	7
Age at diagnosis (days)	Prenatal	30	60	Prenatal	28	9	90	7	34	30	60
Current age (years)	22	19	11	9	6	9	6	2	6	9	15
Sex	Male	Male	Male	Male	Female	Male	Female	Male	Female	Female	Female
*Anthropometric measurements*											
Weight (SD)	+0.06	−2.49	−0.77	+0.48	+0.57	+0.34	N/A	N/A	−0.54	−0.50	−3.29
Height (SD)	−0.92	−1.19	+0.21	+0.33	+2.53	+0.62	N/A	N/A	−0.95	−2.66	N/A
OFD (SD)	N/A	+2.02	−0.90	−0.39	−1.13	+0.35	N/A	N/A	−0.65	−2.39	−2.86 SD
*Metabolic presentation*											
Type of presentation	Classical	Classical	Classical	N/A	Classical	Classical	Classical	Classical	Classical	Classical	Classical
Leucine^a^ (35–270) μmol/L	2047	2418	1850	N/A	1407	571	866	3035	629	552	4000
Valine^a^ (51–325 μmol/L)	771	432	N/A	N/A	548	175	611	780	Normal	Normal	N/A
Isoleucine^a^ (13–135 μmol/L)	543	587	N/A	N/A	175	125	573	375	Normal	Normal	1300
Dietary compliance	Noncompliant	Moderately compliant	Strictly compliant	Strictly compliant	Moderately compliant	Moderately compliant	Compliant	Compliant	Compliant	Strictly compliant	Noncompliant
Metabolic crises before liver transplantation	Frequent^b^	Occasional^b^	Occasional^b^	Absent	Frequent^b^	Frequent^b^	Frequent^b^	Frequent^b^	Frequent^b^	Absent	Frequent^b^
Metabolic crises requiring dialysis	Once	Once	None	None	None	None	Once	Once	None	None	Once
*Neurodevelopment*											
Walk delay	+	+	−	−	−	−	+	−	+	+	++
Abnormal gait	+	+	−	−	−	−	−	−	+	+	+
Speech delay	−	−	−	−	−	−	−	−	−	−	+
Intellectual disability	Moderate–severe ID	Moderate ID	LD	−	−	−	N/A	−	−	LD	Moderate–severe ID
Schooling	Left at 10th grade	Left At 10th grade	2nd grade	2nd grade	Kindergarten	2nd grade	N/A	N/A	2nd grade	transitional kindergarten	none
Seizures	−	+	+	−	−	−	−	−	−	−	+
Behavior	Aggressive	Appropriate	Appropriate	Appropriate	Appropriate	Appropriate	Appropriate	Appropriate	Appropriate	Appropriate	Appropriate
Independence	Partial	Full	Full	Full	Full	Partial	No	No	Partial	Partial	No
Brain MRI	Normal	Normal	N/A	N/A	N/A	N/A	N/A	N/A	Diffuse WM hyperintensity	N/A	N/A

Abbreviations: +, presence of the clinical feature; ++, severe manifestation; −, absence of the feature; ID, intellectual disability; LD, learning difficulty; N/A, not available; OFD, occipitofrontal diameter; SD, standard deviation; WM, white matter; yrs, years.

^a^ Values represent plasma concentrations measured during episodes of acute metabolic crisis.

^b^ Frequent crises were defined as an average of six hospitalizations per year, while occasional crises were defined as one or fewer hospitalizations per year.

### 3.2 Dietary Interventions and Adherence Outcomes

Long‐term management included a protein‐restricted diet with BCAA‐free formulas (1–2 g/kg/day) and regular plasma amino acid monitoring, targeting leucine 76–380 μmol/L and isoleucine/valine 200–400 μmol/L. All patients received supplemental valine and isoleucine to maintain target plasma levels and prevent secondary deficiencies. Dietary compliance was assessed longitudinally based on clinic attendance, caregiver‐reported adherence, and serial plasma BCAA concentrations and was classified as strict (consistently within target ranges), moderate (intermittent deviations), or noncompliant (frequent elevations outside target ranges).

Patients presenting with encephalopathy within the first few days of life, emergency treatment focused on halting protein intake, administering intravenous glucose, and monitoring blood glucose and electrolytes to prevent complications like brain edema and organ dysfunction. Intralipids were provided via parenteral nutrition, and intravenous insulin was administered when blood glucose levels exceeded 250 mg/dL. Acute crises were managed with peritoneal dialysis, followed by leucine‐free enteral feeding with valine and isoleucine supplementation. Thiamin (100 mg/day) was administered without response.

Five participants (45%) received peritoneal dialysis during severe metabolic crises with very high plasma leucine and persistent coma. Thiamine (100 mg/day) was given to those awaiting genetic confirmation, but no thiamine‐responsive phenotypes were observed. Nine participants (81%) were moderately to strictly adherent to dietary management, while two were noncompliant (IV:6 from Family 1 and the participant from Family 9; Table 1). Dietary adherence correlated with plasma BCAA levels within target ranges and fewer or no acute metabolic crises; strict dietary adherence was strongly associated with a reduced frequency, or complete absence, of acute metabolic crises (V2 and V3 in Family 3, and IV:1 in Family 8, Table 1).

### 3.3 Genetic Studies

Among the 10 participants, four homozygous disease‐causing variants were identified. One participant (Family 9, Table 1) declined follow‐up, pedigree analysis, and further genetic testing. Parental DNA was available for all tested cases, confirming biparental inheritance of the variants. Pathogenic *DBT* variants were detected in eight participants (80%), a likely pathogenic *BCKDHA* variant in one participant (10%), and a disease‐causing *BCKDHB* variant in another (10%). All identified variants are predicted to result in loss‐of‐function of the encoded protein. Genotypes, predicted protein consequences, and allele frequencies based on gnomAD v4.1.0 are summarized in Table 2. Six participants from four unrelated families in the same community shared the same pathogenic *DBT* gene variant (p.Met444_Asn445insTer). Interestingly, participant V:2 from Family 7, who also originated from the same community, presented with a distinct splice site variant in the *BCKDHB* gene (NM_183050.4:c.634‐2A>G).

**TABLE 2:** Summary of sequence variants identified in individuals with MSUD.

Patient	Testing method	Gene	Genomic position (GRCh38)	Coding change NM_001918.5	Protein prediction	Zygosity	Inheritance	gnomAD AF	gnomAD AF ME	ClinVar classification	Variant classification^a^
Families 1 and 2	Gene sequencing	*DBT*	chr1:100249791C>T	c.30G>A	p.(Trp10Ter)	HOM	Biparental	1.247e−6	Absent	Pathogenic	Pathogenic
Families 3–6	Gene sequencing	*DBT*	Chr1:100196368‐100196371del	c.1333_1336del	p.(Met444_Asn445insTer)	HOM	Biparental	Absent	Absent	Pathogenic	Pathogenic
Family 7	Exome sequencing	*BCKDHB*	Chr6:80171280A>G	c.634‐2 A>G	Splice acceptor	HOM	Biparental	4.394e−6	Absent	Likely pathogenic	Pathogenic
Family 8	Exome sequencing	*BCKDHA*	Chr19:41410622T>A	c.109‐15T>A	Activation of cryptic splice site and frameshift	HOM	Biparental	1.859e−6	Absent	VUS	Likely pathogenic

Abbreviations: AF, allele frequency in the gnomAD v4 population (accessed April 2025); AF ME, allele frequency in the Middle Eastern subpopulation of gnomAD v4; HOM, homozygous; LP, likely pathogenic; VUS, variant of uncertain significance.

^a^ According to ACMG guidelines [25].

Diagnostic exome sequencing of participant IV:1 from Family 8 identified a likely pathogenic intronic splice site gain variant in *BCKDHA* gene (NM_000709.4:c.109‐15T>A), with a SpliceAI D score of 0.53 (maximum = 1) [26]. The functional significance of the region and the potential impact of the substitution were supported by a CADD score of 18.5 [27], and a marked depletion of intronic variants in the region surrounding the variant site in gnomAD with a complete absence of homozygous intronic variants extending up to 40 bases upstream (5′ direction) from the variant site, and a documented Gnocchi Z score for regional constraint including functional non‐coding annotations of 2.2 (Figure S1a) [28]. Reverse transcription PCR (RT‐PCR) on RNA extracted from the affected individual showed that the splice site variant caused retention of 13 nucleotides in exon 2, producing a frameshift and predicted loss of protein function. (Figure S1b,c). No other MSUD‐related variants were identified, confirming this variant as the likely cause of the clinical phenotype.

### 3.4 Outcome After Liver Transplantation

The results of liver transplantation are summarized in Table 3. Nine participants underwent liver transplantation, with indications including poor metabolic control and diminished quality of life due to dietary restrictions. In eight of the nine cases, the donors were living‐related parents who were obligate carriers of the MSUD‐associated variant. One participant (Family 1, IV:6) is currently awaiting a suitable donor match. All transplanted participants received grafts from related donors, with the age at transplantation varying across the cohort from 6 months to 18 years. Participant and graft survival rates were 100% at a mean follow‐up period of 2.6 years.

**TABLE 3:** Summary of clinical outcomes in MSUD patients after liver transplantation.

Patient	Age at transplantation (years)	Current age	Donor	Graft survival	Surgical complications	Patient alive	Metabolic crisis after transplantation	Neurodevelopmental outcome after transplantation				
								Motor	Cognition	Behavior	Independence	Education
Family 2, IV:3	18	19	Father	Yes	Biliary obstruction managed with stenting	Yes	No	Spastic gait	Delayed	Appropriate	Full	Left at 10th grade
Family 3, V:2	8	11	Father	Yes	Bile leak/diaphragmatic hernia	Yes	No	Normal	Learning difficulty	Appropriate	Partial	2nd grade
Family 3, V:3	6	9	Mother	Yes	No	Yes	No	Normal	Normal	Appropriate	Full	2nd grade
Family 4, VI:2	3.5	6	Mother	Yes	No	Yes	No	Normal	Normal	Appropriate	Full	Kindergarten
Family 5, VI:2	4.5	9	Paternal uncle	Yes	EBV viral infection	Yes	No	Normal	Normal	Appropriate	Partial	2nd grade
Family 6, IV:1	3	6	Father	Yes	No	Yes	No	Spastic gait	Normal	Appropriate	No	None
Family 6, IV:2	0.5	2	Mother	Yes	No	Yes	No	—	Normal	Appropriate	No	Not yet
Family 7, V:2	4	6	Mother	Yes	No	Yes	No	Spastic gait	Normal	Appropriate	Partial	2nd grade
Family 8, IV:1	5.5	9	Mother	Yes	Pneumonia	Yes	No	Improvement in spastic gait	Normal	Appropriate	Partial	Transitional kindergarten

Immediate post‐transplant complications were observed in 44% of Participants, yet all proved self‐limiting. One individual (IV:3 from Family 2) developed biliary obstruction necessitating stent placement, while another (V:2 from Family 3) experienced a bile leak complicated by diaphragmatic hernia, which was successfully repaired surgically. A third Participant (VI:2 from Family 5) contracted Epstein–Barr virus infection, resolving fully with conservative management, and a fourth (IV:1, Family 8) developed postoperative pneumonia that responded to standard antimicrobial therapy. Long‐term outcomes were notably positive. According to patient and family reports, the immediate post‐transplant course abroad included approximately 3 weeks of inpatient care with gradual reintroduction of oral feeding and regular monitoring of plasma BCAA levels. Patients then completed a total of 2 months of clinical follow‐up before discharge, at which point all were able to resume an unrestricted, age‐appropriate table‐food diet. All transplanted participants transitioned to a normal diet with improved leucine tolerance, and none required hospitalization or experienced acute metabolic crises during the two‐year follow‐up. No post‐transplant decline in motor, speech, or cognitive development was observed. In fact, three participants (V:2 and V:3 from Family 3; VI:2 from Family 5) showed marked developmental improvements, achieving milestones comparable to age‐matched peers (Table 3).

## 4 Discussion

This study is the first to comprehensively describe the clinical, molecular, and therapeutic landscape of MSUD in the Palestinian population. We analyzed 11 participants from nine unrelated families, identifying a high prevalence of severe early‐onset disease, with 91% presenting with classical MSUD with acute metabolic crises in the first days of life and 54.5% exhibiting significant developmental and cognitive impairments. Genetic analyses revealed recurrent and novel pathogenic variants, including a shared *DBT* founder variant in six participants from four unrelated families, as well as distinct *BCKDHA* and *BCKDHB* variants in other cases. Therapeutically, five participants (45%) required peritoneal dialysis during acute crises, while long‐term management with protein‐restricted, BCAA‐free diets achieved plasma amino acid targets in nine participants (81%) with strict adherence, markedly reducing the frequency of metabolic decompensation. Liver transplantation was performed in nine participants, with 100% graft and patient survival over a mean follow‐up of 2.6 years; all transplanted individuals transitioned to a normal diet and remained free of acute crises, and three demonstrated notable developmental gains.

Founder genetic variants were identified in multiple unrelated families (Families 3–6), reflecting a high degree of genetic homogeneity likely driven by population structure and marriage patterns. Notably, even within a community where a founder variant is prevalent, novel pathogenic MSUD‐associated variants were identified in other families (e.g., Family 7), further illustrating the emergence of novel disease‐causing variants. Currently, there is no national newborn screening for MSUD; however, the emergence of novel pathogenic variants necessitates that any future screening strategies integrate both biochemical and molecular approaches to ensure comprehensive detection, particularly in newborns presenting with acute encephalopathy. The lack of such screening has delayed diagnoses, resulting in infant deaths, prolonged metabolic crises, encephalopathy, poor neurodevelopment, and often postponed liver transplantation. Liver transplantation, when performed, effectively stabilizes BCAA metabolism and prevents acute crises. In this study, all transplanted participants transitioned to a normal diet, showed improved leucine tolerance, and remained hospitalization‐free during follow‐up (Table 3).

Neurodevelopmental outcomes in our cohort were strongly influenced by age at diagnosis, dietary adherence, timing of liver transplantation, and prior metabolic crises. Consistent with previous reports, liver transplantation alone does not reverse existing neurological damage [16, 29], and long‐term studies have shown limited impact on cognitive recovery [21]. In contrast, our findings reinforce that early, integrated intervention is critical: one child diagnosed prenatally and managed with strict dietary therapy before transplantation achieved normal cognitive, motor, and speech development, while another transplanted at six months reached full developmental milestones by age two. These cases highlight that timely diagnosis and proactive management can prevent irreversible neurological injury and align with evidence from recent meta‐analyses emphasizing early detection as the strongest predictor of favorable neurocognitive outcomes [30]. While larger, long‐term studies are needed, this study is the first to show that early diagnosis, dietary management, and timely liver transplantation can prevent neurological damage and support favorable development, even in populations with high‐variant prevalence and limited access to specialized care.

## 5 Conclusions

Our findings support an integrated MSUD management approach, combining metabolic screening, founder‐variant mapping, and timely interventions like liver transplantation, particularly in high‐prevalence populations, to enable early diagnosis and improve outcomes.

## Author Contributions

R.K.‐N., H.H.‐A., J.Z., M.A., and I.D. contributed to data acquisition and interpretation. R.K.‐N. and I.D. were responsible for the initial conception and design of the study. I.D. also oversaw patient care and supervised data collection. R.K.‐N. and H.H.‐A. drafted the original manuscript. All authors participated in data interpretation, manuscript editing and review, and provided critical input throughout the revision process. All authors have read and approved the final manuscript for submission.

## Funding

The authors have nothing to report.

## Ethics Statement

Ethical approval was granted by the Institutional Review Board of the Arab American University (approval numbers 2022/C/10/N and 2024/A/3/C). This study was conducted in accordance with the Declaration of Helsinki and the protocols of the local ethics committee.

## Consent

Informed consent for comprehensive clinical and biochemical phenotyping, as well as publication of the results, was obtained from all participants and/or their legal guardians, and assent was obtained from the patients where appropriate.

## Conflicts of Interest

The authors declare no conflicts of interest.

## Supporting information


**Figure S1:** Genomic and transcript‐level characterization of the intronic variant NM_000709.4:c.109‐15 T>A in the *BCKDHA* gene. (a) The shaded region highlights a local depletion of intronic variation in Genome Aggregation (gnomAD) Database, v4.1.0, suggesting evolutionary constraint of potential functional relevance. The position of the c.109‐15 T>A variant is indicated by an arrow within this conserved segment. (b) Schematic representation of the *BCKDHA* gene structure, showing exon–intron boundaries. Exons are depicted as boxes, with the variant mapped relative to the gene structure (arrow). (c) Sanger sequencing of reverse‐transcribed RNA from the patient sample with homozygous c.109‐15 T>A reveals the insertion of an additional 14 nucleotides, highlighted in yellow, in the mRNA transcript between exon 1 and exon 2.

## Data Availability

The data that support the findings of this study are available on request from the corresponding author. The data are not publicly available due to privacy or ethical restrictions.
