# Supplementary material for: Clinical Profiles, Genetic Variants, and Neurodevelopmental Outcomes Following Liver Transplantation in Maple Syrup Urine Disease: A Study From Palestine
Source: JIMD Rep. 2026 Feb 22;67(2):e70077. doi: 10.1002/jmd2.70077 (PMC12928098; doi:10.1002/jmd2.70077)
Supplement: Supplementary file 1 — Figure S1: Genomic and transcript‐level characterization of the intronic variant NM_000709.4:c.109‐15 T>A in the BCKDHA gene. (a) The shaded region highlights a local depletion of intronic variation in Genome Aggregation (gnomAD) Database, v4.1.0, suggesting evolutionary constraint of potential functional relevance. The position of the c.109‐15 T>A variant is indicated by an arrow within this conserved segment. (b) Schematic representation of the BCKDHA gene structure, showing exon–intron boundaries. Exons are depicted as boxes, with the variant mapped relative to the gene structure (arrow). (c) Sanger sequencing of reverse‐transcribed RNA from the patient sample with homozygous c.109‐15 T>A reveals the insertion of an additional 14 nucleotides, highlighted in yellow, in the mRNA transcript between exon 1 and exon 2. [file JMD2-67-e70077-s001.pptx]

## Slide 1
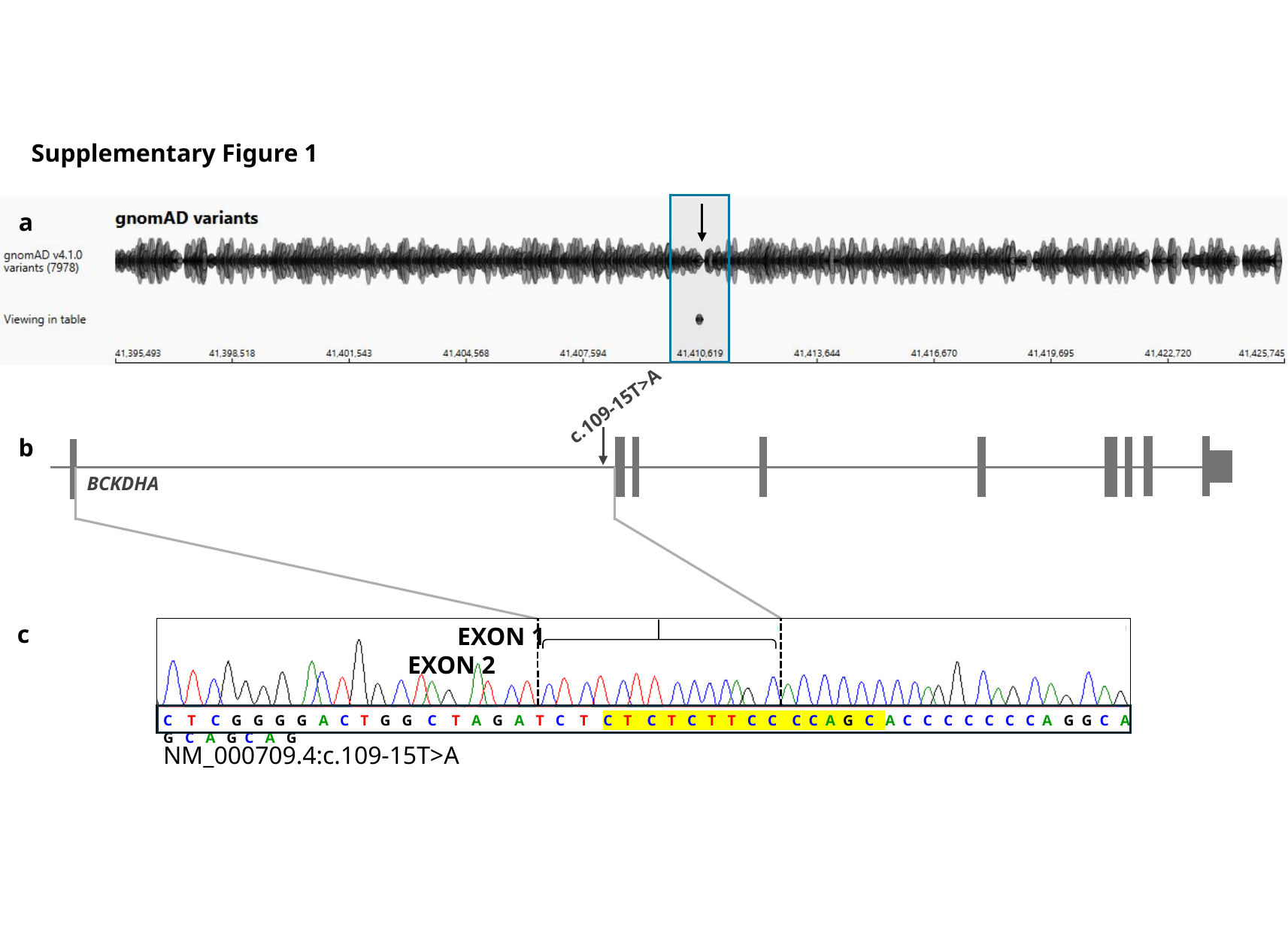

Supplementary Figure 1
a
c.109-15T>A
b
BCKDHA
c
 EXON 1 EXON 2
C T C G G G G A C T G G C T A G A T C T C T C T C T T C C C C A G C A C C C C C C C A G G C A G C A G C A G
NM_000709.4:c.109-15T>A

## Slide 2
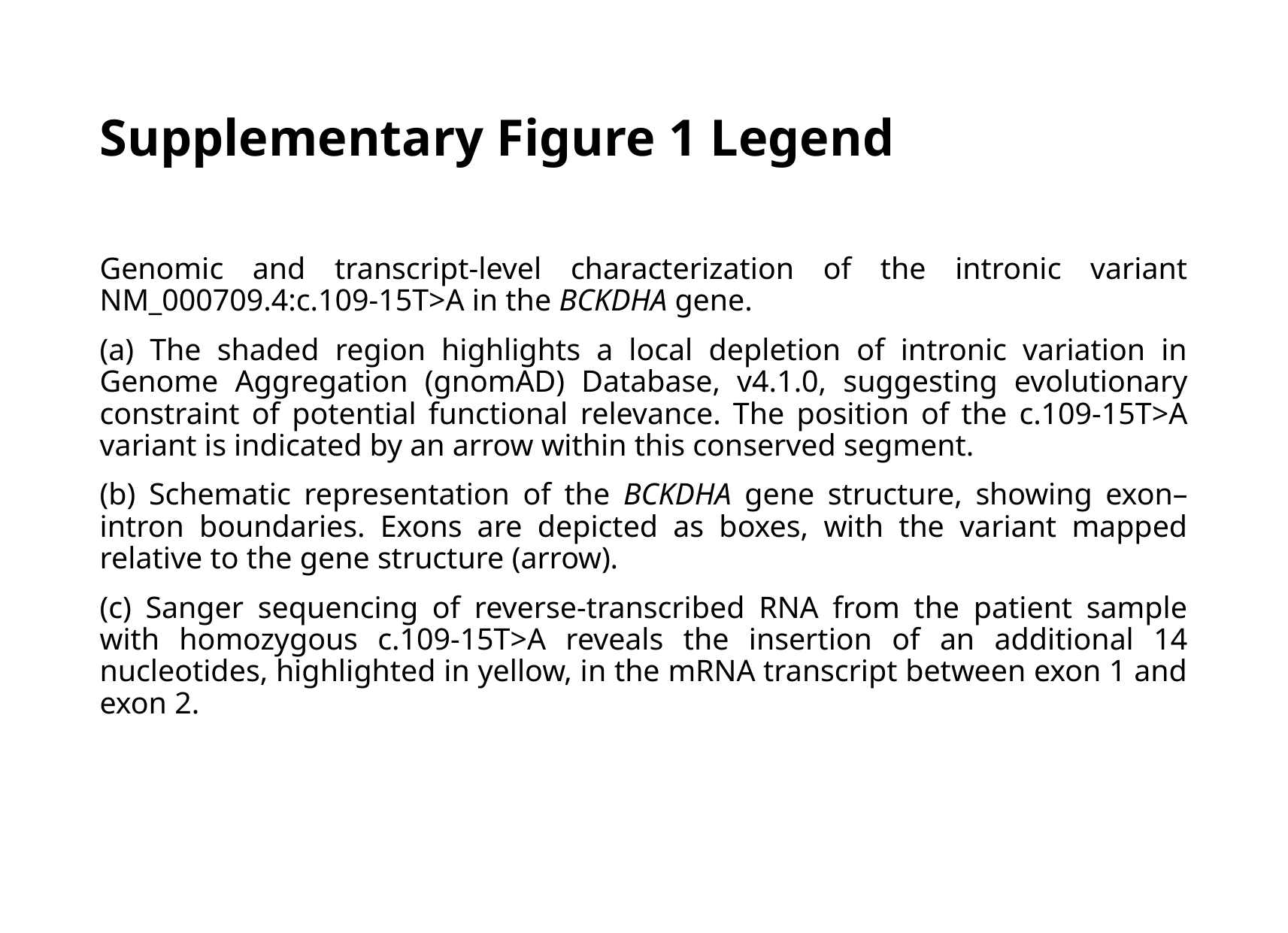

# Supplementary Figure 1 Legend
Genomic and transcript-level characterization of the intronic variant NM_000709.4:c.109-15T>A in the BCKDHA gene.
(a) The shaded region highlights a local depletion of intronic variation in Genome Aggregation (gnomAD) Database, v4.1.0, suggesting evolutionary constraint of potential functional relevance. The position of the c.109-15T>A variant is indicated by an arrow within this conserved segment.
(b) Schematic representation of the BCKDHA gene structure, showing exon–intron boundaries. Exons are depicted as boxes, with the variant mapped relative to the gene structure (arrow).
(c) Sanger sequencing of reverse-transcribed RNA from the patient sample with homozygous c.109-15T>A reveals the insertion of an additional 14 nucleotides, highlighted in yellow, in the mRNA transcript between exon 1 and exon 2.
